# Liver metastases 2 years after resection of a very-low-risk duodenal gastrointestinal stromal tumor: a case report

**DOI:** 10.1186/s40792-022-01551-1

**Published:** 2022-10-10

**Authors:** Junya Mita, Kazuhiro Tada, Yusuke Kuboyama, Shoji Hiroshige, Shun Nakamura, Junichi Takahashi, Kazuhito Sakata, Hiroshi Mizuuchi, Taro Oba, Fumitaka Yoshizumi, Kentaro Iwaki, Hideya Takeuchi, Kiyoshi Kajiyama, Kengo Fukuzawa

**Affiliations:** 1 Department of Surgery, Oita Red Cross Hospital, 3-2-37 Chiyomachi, Oita-Shi, Oita, Japan; 2 Department of Pathology, Oita Red Cross Hospital, 3-2-37 Chiyomachi, Oita-Shi, Oita, Japan

**Keywords:** GIST, Imatinib, Mitotic rate

## Abstract

**Background:**

Gastrointestinal stromal tumors (GISTs) are rare mesenchymal tumors, but are the most common mesenchymal tumors of the gastrointestinal tract. The risk classification of GISTs is based on the tumor size, mitotic index, tumor site, and presence of tumor rupture. Recurrence in the very-low-risk group is extremely rare. We herein report a case of liver metastases 2 years after resection of a very-low-risk duodenal GIST.

**Case presentation:**

A 57-year-old woman presented to the hospital for evaluation of melena. Esophagogastroduodenoscopy showed bleeding from the exposed blood vessels at the top of a submucosal tumor approximately 20 mm in size located in the second (descending) part of the duodenum, and the bleeding was controlled with electrocoagulation. A GIST was suspected, and the patient underwent wedge resection of the duodenum. The resected specimen contained a 16- × 12-mm (< 20-mm) white submucosal tumor composed of spindle cells with a mitotic count of 4 per 50 high-power fields, and a histologically negative margin was achieved. Immunochemical analysis revealed positive tumor staining for c-kit protein and alpha-smooth muscle actin and negative staining for CD34, desmin, and S-100 protein. Therefore, the tumor was diagnosed as a very-low-risk duodenal GIST based on the Fletcher classification and modified Fletcher classification (Joensuu classification). The postoperative course was uneventful, and the patient was discharged on postoperative day 11. At the follow-up visit 2 years postoperatively, contrast-enhanced computed tomography revealed liver tumors in S8 and S6 measuring 26 × 24 and 10 × 10 mm, respectively. Both lesions showed peripheral dominant hyperenhancement with hypoenhancement inside, indicating tissue degeneration within the tumors. These imaging findings closely resembled those of the duodenal GIST. Hence, the patient was diagnosed with liver metastases of GIST 2 years postoperatively. She was subsequently started on treatment with 400 mg of imatinib. At the time of this writing (2 months after diagnosis), the patient was clinically well and asymptomatic and was continuing imatinib therapy.

**Conclusions:**

Recurrence of very-low-risk GISTs is extremely rare. Even a small GIST with low mitotic activity can never be considered completely benign, and long-term follow-up is necessary.

## Background

Gastrointestinal stromal tumors (GISTs) are rare mesenchymal tumors, but they are the most common mesenchymal tumors of the gastrointestinal tract. The risk classification of GISTs is based on the tumor size, mitotic index, tumor site, and presence of tumor rupture. Recurrence of very-low-risk GISTs is quite rare. We herein present an extremely rare case of recurrence of a very-low-risk duodenal GIST.

## Case presentation

**Fig. 1:**
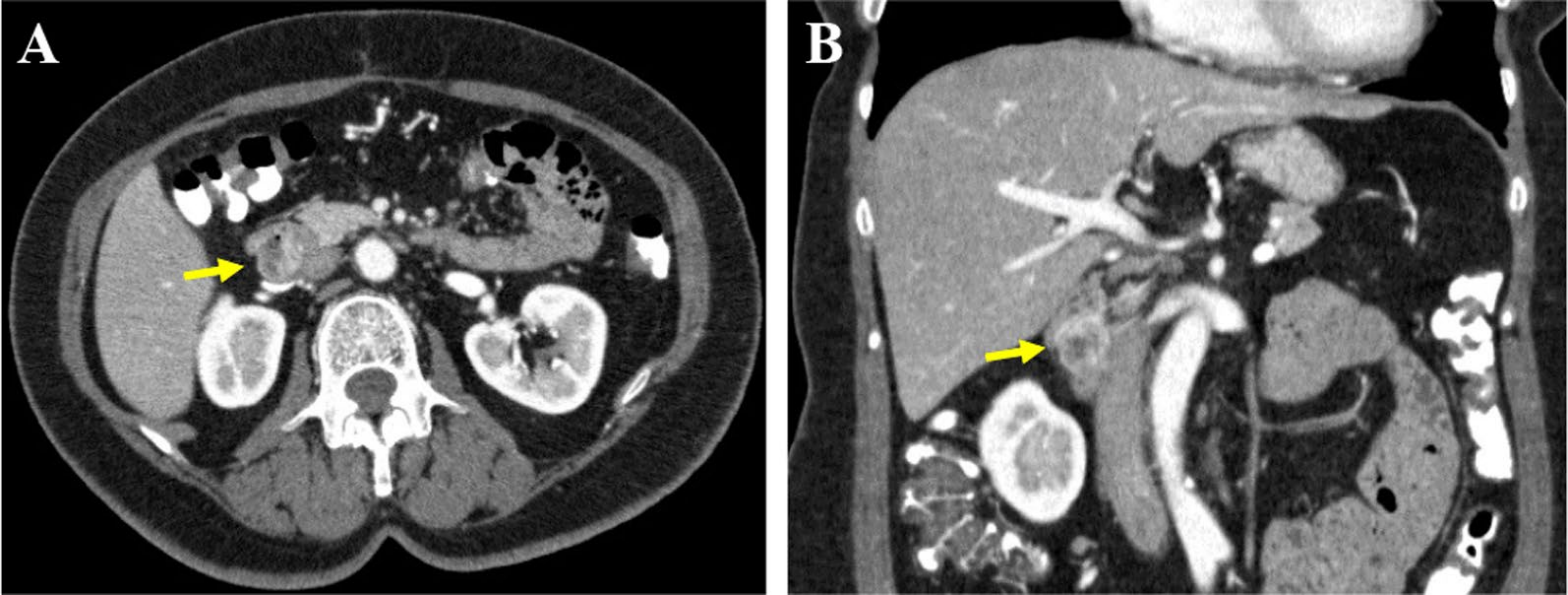
**A**, **B** Contrast-enhanced computed tomography at the first visit. Extravasation was seen from a 20-mm mass in the second part of the duodenum (arrow)

**Fig. 2:**
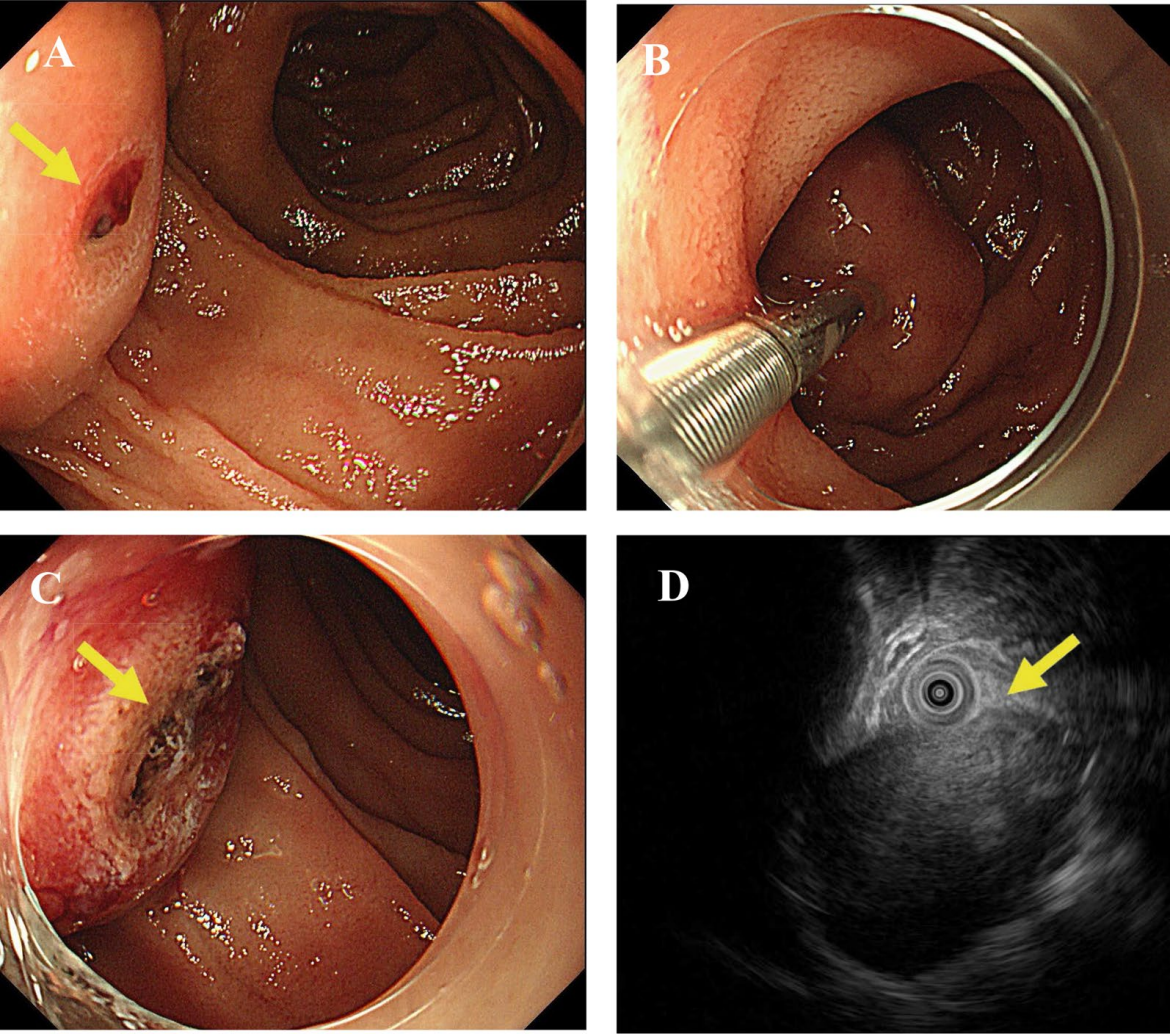
**A** Esophagogastroduodenoscopy showed bleeding from the exposed blood vessels at the top of the 20-mm submucosal tumor in the second part of the duodenum (arrow). **B**, **C** The bleeding was successfully controlled with electrocoagulation (arrow). **D** Endoscopic ultrasonography showed a 16.6-mm hypoechoic lesion originating from the fourth layer of the duodenal wall (arrow)

**Fig. 3:**
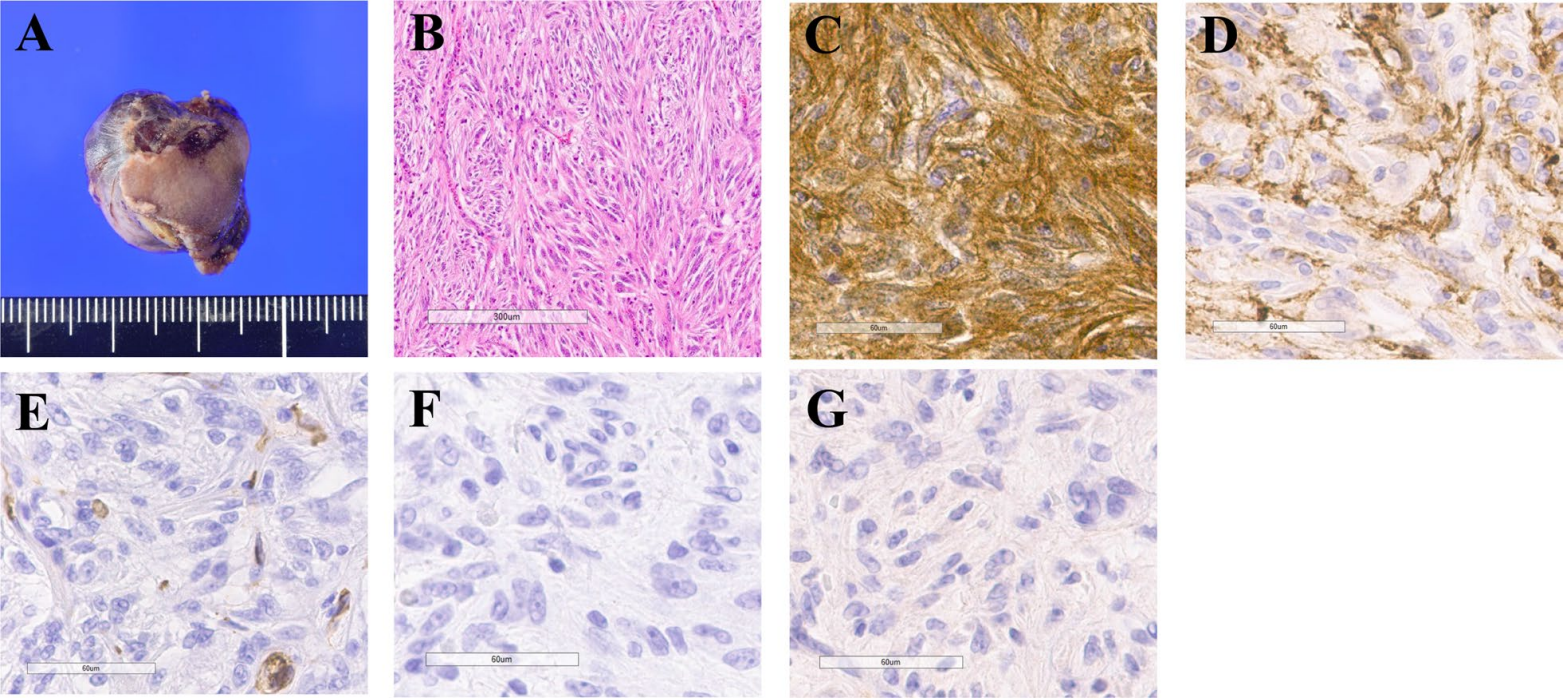
**A** Gross inspection of the specimen showed a firm white tumor measuring 16 × 12 mm. **B** Histopathological evaluation showed that the tumor was composed of spindle cells with a mitotic count of 4 per 50 high-power fields. Immunochemical analysis revealed positive tumor staining for **C** c-kit protein and **D** alpha-smooth muscle actin and negative staining for **E** CD34, **F** desmin, and **G** S-100 protein

A 57-year-old woman with type 2 diabetes mellitus presented to another hospital for evaluation of melena. Her clinical evaluation findings were unremarkable; however, laboratory examination demonstrated a low hemoglobin level of 9.9 g/dL. All other routine hematological and biochemical profiles were within the reference ranges. Contrast-enhanced computed tomography (CT) showed extravasation arising from a mass of high signal intensity in the second part of the duodenum (Fig. 1). Esophagogastroduodenoscopy showed bleeding from the exposed blood vessels at the top of the submucosal tumor, which was approximately 20 mm in size and located in the second (descending) part of the duodenum; the bleeding was controlled with electrocoagulation (Fig. 2A–C). Endoscopic ultrasonography showed a 16.6-mm hypoechoic lesion arising from muscularis propria layer on the posterior wall of the descending duodenum (Fig. 2D). Fine needle aspiration biopsy was not performed because of the risk of rebleeding. A GIST was suspected, and the patient was referred to our hospital for surgical intervention. The tumor was detected on the posterior wall of the descending duodenum, protruding outside the serosal surface. After mobilization from the first to third portion of the duodenum using the Kocher maneuver, wedge resection was performed, and the duodenal wall was closed with sutures. The operation time was 110 min, and the blood loss volume was 50 mL. The resected specimen contained a 16- × 12-mm white submucosal tumor. Histopathological evaluation showed that the tumor was composed of spindle cells with a mitotic count of 4 per 50 high-power fields (HPFs) and that a histologically negative margin had been achieved. Hemorrhage and necrosis of the tumor were also observed. Immunochemical analysis revealed positive tumor staining for c-kit protein and alpha-smooth muscle actin and negative staining for CD34, desmin, and S-100 protein (Fig. 3). Therefore, the tumor was diagnosed as a very-low-risk duodenal GIST based on the Fletcher classification and the modified Fletcher classification (Joensuu classification). Furthermore, the tumor was classified as no-risk according to the Miettinen classification. The postoperative course was uneventful, and the patient was discharged on postoperative day 11.

**Fig. 4:**
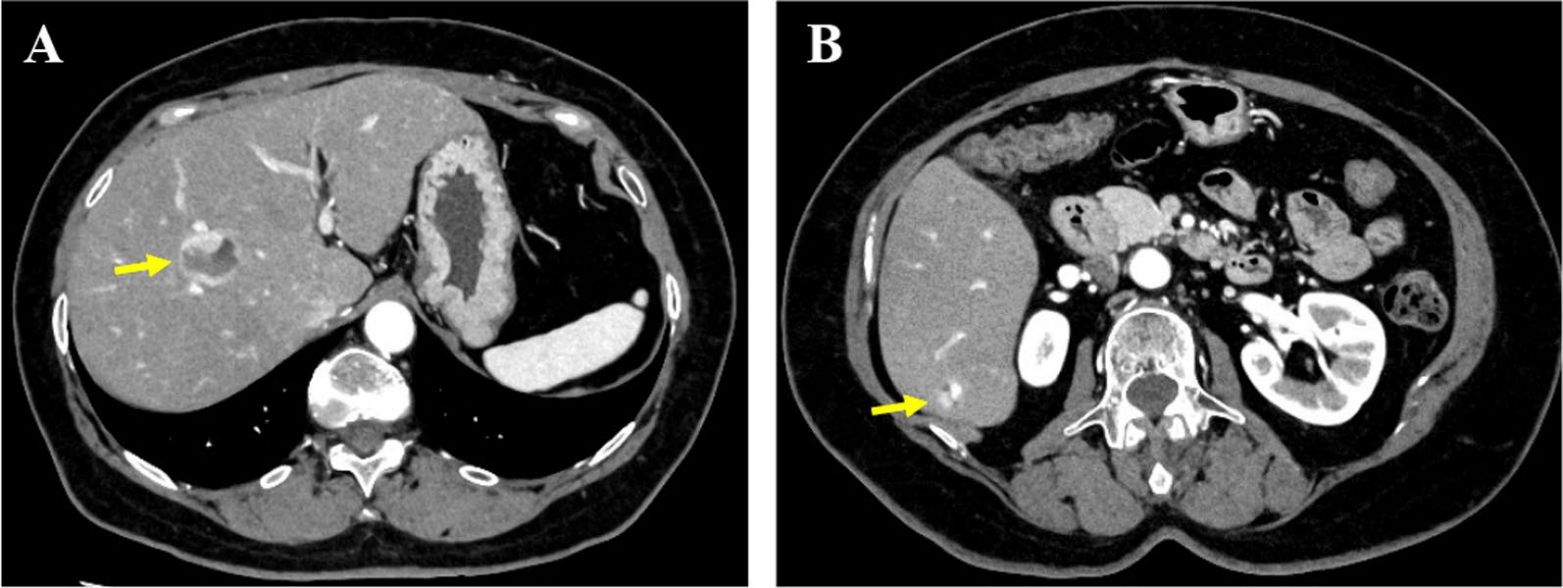
Contrast-enhanced computed tomography at the follow-up visit 2 years postoperatively. **A** A 26- × 24-mm mass in S8 (arrow) and **B** a 10- × 10-mm mass in S6 (arrow) were shown as hypervascular lesions in the early phase on contrast-enhanced computed tomography

Follow-up included physical examination every 3 months, and abdominal ultrasonography and CT scan every 6 months. At the follow-up visit 2 years postoperatively, abdominal ultrasound showed two heterogeneous hyperechoic lesions in the liver: a 25-mm mass in S8 and a 10-mm mass in S6. There was no increase in the tumor markers for hepatocellular carcinoma or gastrointestinal cancer. Contrast-enhanced CT revealed liver tumors in S8 and S6 measuring 26 × 24 mm and 10 × 10 mm, respectively (Fig. 4). Both lesions showed peripheral dominant hyperenhancement with hypoenhancement inside, indicating tissue degeneration within the tumors. There was no local recurrence in the duodenum after surgery. Magnetic resonance imaging showed hypervascular tumors with restricted diffusion and low apparent diffusion coefficients; they contained tissue degeneration within them, and they measured 25 × 21 mm and 10 × 8 mm in S8 and S6, respectively. The imaging findings of both tumors closely resembled those of the duodenal GIST. Hence, the patient was diagnosed with liver metastases of GIST 2 years postoperatively. She was subsequently started on treatment with 400 mg of imatinib. At the time of this writing (2 months after diagnosis), the patient was clinically well and asymptomatic and was continuing the imatinib therapy.

## Discussion

GISTs are rare mesenchymal tumors and can arise anywhere from the interstitial cells of Cajal at the submucosal and myenteric plexus of the gastrointestinal tract. They are the most common mesenchymal tumors of the gastrointestinal tract; approximately 60% of them originate in the stomach, followed by the small bowel (30%), rectum (5%), and duodenum (< 5%) [1, 2]. The symptoms of GISTs are variable, and the clinical presentation includes diffuse abdominal pain, bleeding, fever, and obstruction. Histologically, GISTs are often divided into spindle cell types and epithelioid cell types. They have a characteristic immunohistochemical pattern in that CD117, which is part of the c-kit tyrosine kinase receptor, is positive in > 95% of cases. Expression of CD34 occurs in > 80% of GISTs, and alpha-smooth muscle actin is demonstrable in about 25% [3, 4]. The standard treatment for GISTs is surgical resection with negative margins; however, the optimal margin width has not been defined. Local recurrence or metastasis occurs in approximately 40% of cases after curative resection, with liver metastasis being the main recurrence pattern of GISTs [5]. The kit inhibitor imatinib is the standard first-line therapy for recurrence following resection of primary GISTs.

Duodenal GISTs constitute < 5% of GISTs and mostly occur in the second part of the duodenum, followed by the third, fourth, and first part [1]. The most common clinical presentation of duodenal GISTs is bleeding or abdominal pain [2]. Unlike other types of GISTs, the optimal surgical procedure for duodenal GISTs has not been definitively determined. In the stomach, the most common site of GISTs, limited resection is technically simple in most cases; in the duodenum, however, local resection can be more complicated. Pancreatoduodenectomy may even be performed when the tumor is located in the descending part of the duodenum or involves the ampulla of Vater and pancreatic head. Nillson et al. [6] reported that low-risk gastric GISTs carry a 1.9% risk of recurrence, whereas low-risk duodenal GISTs have an 8.3% recurrence rate. Duodenal GISTs are often large at the time of diagnosis and tend to be located in the muscle layer and grow into the submucosa, resulting in both ulceration and hemorrhage. These factors are related to a higher malignant potential than that of gastric GISTs, though no specific gene products have been identified to account for the prognostic differences [7, 8].

According to the literature, several risk stratifications have been proposed: the Fletcher classification, the modified Fletcher classification (Joensuu classification), and the Miettinen classification. The Fletcher classification is based on tumor size and mitotic index. It subdivides tumors into very low risk (tumor size of < 2 cm and mitotic count of < 5 per 50 HPFs), low risk (tumor size of 2–5 cm and mitotic count of < 5 per 50 HPFs), intermediate risk (tumor size of < 5 cm and mitotic count of 6–10 per 50 HPFs, or tumor size of 5–10 cm and mitotic count of < 5 per 50 HPFs), and high risk (tumor size of > 5 cm and mitotic count of > 5 per 50 HPFs, tumor size of > 10 cm and any mitotic index, or tumor of any size and mitotic count of > 10 per 50 HPFs) [9]. The modified Fletcher classification includes the tumor site and presence of rupture as additional variables [10]. The Miettinen classification is based on tumor size, mitotic index, and location (Table 1) [11]. In the present case, the tumor in the duodenum was 16 mm in size and had a mitotic count of 4 per 50 HPFs; it was therefore classified as very low risk according to the Fletcher classification and the modified Fletcher classification and as no risk based on the Miettinen classification. In patients who have undergone complete resection of no-risk, very-low-risk, or low-risk GISTs, follow-up by abdominal CT is recommended every 6 months for 5 years after surgery [12]. Recurrence in the very-low-risk group is extremely rare, and even in the low-risk group, recurrence is quite rare (2.4%) after complete surgical removal [6]. To the best of our knowledge, only one other case report of postoperative recurrence of a very-low-risk GIST has been published to date [13]. In that case, suture-line recurrence at the gastrojejunal anastomosis appeared 8 years after resection; this might be considered local recurrence after surgery. In our case, the liver metastases appearing 2 years after surgery were classified as distant metastases, and this could be the first report of distant metastases of a very-low-risk GIST after radical resection. In addition to the prognostic factors used in the classifications, several other prognostic factors have been reported. From a clinical and histological viewpoint, tumor necrosis, hemorrhage, mucosal ulceration, and vascular invasion are associated with a poor outcome [14, 15]. The CT findings that suggest a malignant potential include a lesion larger than 11.1 cm, an irregular surface, an unclear boundary, the presence of invasion, heterogeneous enhancement, and wall invasion of other organs [16]. In our case, although categorized as very low risk, the GIST had several other malignant features such as heterogenous enhancement, hemorrhage, and tumor necrosis. The patient had not received adjuvant chemotherapy as there was no evidence of its effect on very-low-risk GISTs [17]. The benefit of adjuvant chemotherapy for very-low-risk GISTs with those other malignant features remains unclear. Further data accumulation and its analysis could help to assess whether adjuvant chemotherapy should be given in such cases.

**Table 1:** Miettinen classification [11]

Mitotic counts/50 HPFs	Size (cm)	Stomach (predicted malignant potential)	Ileum (predicted malignant potential)	Duodenum (predicted malignant potential)	Colon (predicted malignant potential)
≤ 5	≤ 2.0	None	None	None	None
≤ 5	2.1–5.0	Very low	Low	Low	Low
≤ 5	5.1–10.0	Low	Moderate	Insufficient data	Insufficient data
≤ 5	> 10.0	Moderate	High	None	High
> 5	≤ 2.0	None	High	None	High
> 5	2.1–5.0	Moderate	High	High	High
> 5	5.1–10.0	High	High	Insufficient data	Insufficient data
> 5	> 10.0	High	High	High	High

Besides the size, mitotic activity, and location of the tumor, several other factors are also related to the malignant potential of GISTs. Therefore, even a small duodenal GIST with low mitotic activity can never be considered as entirely benign, and long-term follow-up is still important.

## Conclusions

We have herein reported an extremely rare case of recurrence of a very-low-risk GIST. Even subtle GISTs can never be considered as truly benign, and long-term follow-up is necessary.

## Data Availability

The authors declare that all the data in this article are available within the article.

## Abbreviations

GIST: Gastrointestinal stromal tumors
EGD: Esophagogastroduodenoscopy
HPF: High-power fields
SMA: Smooth muscle actin
CE-CT: Contrast-enhanced computed tomography
Hb: Hemoglobin
MRI: Magnetic resonance imaging
HPFs: High-power fields
